# Deep Learning Model for Predicting Rhythm Outcomes after Radiofrequency Catheter Ablation in Patients with Atrial Fibrillation

**DOI:** 10.1155/2022/2863495

**Published:** 2022-09-10

**Authors:** Dae-In Lee, Mi-Jung Park, Jee-Woo Choi, Seung Park

**Affiliations:** ^1^Department of Cardiology, Chungbuk National University Hospital, Cheongju-si, Chungcheongbuk-do 28644, Republic of Korea; ^2^Medical AI Research Team, Chungbuk National University Hospital, Cheongju-si, Chungcheongbuk-do 28644, Republic of Korea; ^3^Biomedical Engineering, Chungbuk National University Hospital, Cheongju-si, Chungcheongbuk-do 28644, Republic of Korea

## Abstract

Current guidelines on atrial fibrillation (AF) emphasized that radiofrequency catheter ablation (RFCA) should be decided after fully considering its prognosis. However, a robust prediction model reflecting the complex interactions between the features affecting prognosis remains to be developed. In this paper, we propose a deep learning model for predicting the late recurrence after RFCA in patients with AF. Aiming to predict the late recurrence (LR) of AF within 1 year after pulmonary vein isolation, we designed a multimodal model based on the multilayer perceptron architecture. For quantitative evaluation, we conducted 4-fold cross-validation on data from 177 AF patients including 47 LR patients. The proposed model (area under the receiver operating characteristic curve-AUROC, 0.766) outperformed the acute patient physiologic and laboratory evaluation (APPLE) score (AUROC, 0.605), CHA_2_DS_2_-VASc score (AUROC, 0.595), linear regression (AUROC, 0.541), logistic regression (AUROC, 0.546), extreme gradient boosting (AUROC, 0.608), and support vector machine (AUROC, 0.638). The proposed model exhibited better performance than clinical indicators (APPLE and CHA_2_DS_2_-VASc score) and machine learning techniques (linear regression, logistic regression, extreme gradient boosting, and support vector machine). The model will support clinical decision-making for selecting good responders to the RFCA intervention.

## 1. Introduction

Radiofrequency catheter ablation (RFCA) is accepted as the first-line therapy for patients with symptomatic atrial fibrillation (AF) refractory to antiarrhythmic drugs [1], since Haïssaguerre et al. suggested it as a treatment modality [2]. However, the benefits of RFCA in patients with AF are frequently offset by late recurrence (LR) after the procedure [3]. Moreover, various attempts to modify the atrial substrate, including linear ablation lesion set, ablation targeting rotor, or complex fractionated atrial electrogram, do not demonstrate superiority to pulmonary vein isolation [4, 5]. Therefore, the most recent treatment guidelines for AF recommend assessing the benefit to patients for ensuring a high probability of success after RFCA [1].

Although observational studies have suggested the duration of AF, age, left atrium (LA) size, renal function, and other factors as predictors of LR [6–9], no single factor can accurately predict recurrence after AF ablation [1]. To improve prediction, various models providing indicators such as the CHA_2_DS_2_-VASc score [10, 11] and acute patient physiologic and laboratory evaluation (APPLE) score [12] have been developed. However, these models have shown modest performance [13], as they are based on simple linear regression where each risk factor is assigned one or two points and the sum represents the final score.

Recently, machine learning (ML) methods have been proposed to analyze high-order interactions between different features [14, 15]. For instance, a predictive model based on a support vector machine (SVM) showed an area under the receiver operating characteristic curve (AUROC) of 0.75 for predicting LR within 1 year after RFCA, by considering the AF type (paroxysmal vs. persistent), previous ablation procedure, LA volume, and epicardial fat volume as inputs [16]. In addition, deep learning methods that automatically extract hierarchical features have outperformed traditional ML methods. A recent study [35] employed convolutional neural networks to predict LR from the N-terminal probrain natriuretic peptide, paroxysmal AF, LA appendage volume, and LA volume.

The multilayer perceptron (MLP) technique which analyzes complex nonlinear relations between input features has demonstrated promising performance in various medical applications [18–20]. Therefore, we proposed an MLP-based model for predicting rhythm outcomes after RFCA in patients with AF and compared our model with conventional prediction models and other ML approaches.

## 2. Methods

### 2.1. Study Population and Ethical Statement

We analyzed consecutive patients with AF who underwent RFCA at Chungbuk National University Hospital (CBNUH) from February 2017 to October 2020. All the patients were over 18  years old and underwent their first RFCA. Exclusion criteria included patients with repeated RFCA, with substrate modification lesion sets (e.g., ablation of complex fractionated atrial electrogram or linear ablation), with a follow up period below 1 year, and with missing values in study features. This study was approved by the Institutional Review Board of CBNUH (approval no. 2021-12-009-001). As this was a retrospective observational study, the requirement for informed consent was waived. This study was conducted in accordance with the Declaration of Helsinki.

### 2.2. Preprocedural Preparation and Evaluations

Class I or III antiarrhythmic drugs were discontinued at least half-lives of five times before RFCA. Direct oral anticoagulants were not interrupted during the periprocedural period. One day before the procedure, transthoracic echocardiography and transesophageal echocardiography were acquired from the patients. In addition, the following echocardiographic parameters were collected for this study: left ventricular ejection fraction (LVEF), left ventricle mass index, and LA anterior-posterior diameter. The estimated glomerular filtration rate (eGFR) was also evaluated 1 day before the procedure.

### 2.3. Radiofrequency Catheter Ablation

RFCA was performed under sinus rhythm at our institution, except when AF recurred immediately after cardioversion. Three-dimensional mapping of the LA was constructed using the EnSite NavX/Velocity system (St. Jude Medical, St. Paul, MN, USA). Circumferential pulmonary vein isolation around the antrum of the ipsilateral pulmonary veins was performed using an irrigated TactiCath Quartz or TactiCath TM Contract Force ablation catheter (St. Jude Medical) with a maximum power of 25–40 W. Radiofrequency energy with contact force above 10–20 g was applied in each ablation lesion point until the force-time integral exceed 400 gs. After verifying the electrical isolation of the four pulmonary veins with a bidirectional block, the existence of a nonpulmonary vein trigger was assessed by cardioversion for AF evoked by rapid atrial pacing under isoproterenol infusion.

### 2.4. Clinical Follow-Up

Intake of class I or III antiarrhythmic drugs continued until 3 months after catheter ablation. The rhythm status was assessed by surface electrocardiography (EKG) and Holter monitoring at 2 weeks and 1, 2, 3, 6, 9, and 12 months after discharge. In addition, whenever a patient felt symptoms, EKG and Holter monitoring were performed in our institution. Anticoagulants were prescribed to all patients up to 3 months after discharge, and they were selectively prescribed according to the CHA_2_DS_2_-VASc score afterward.

### 2.5. Definitions

The endpoint of this study was the LR of sustained atrial tachyarrhythmia within 1 year after RFCA. Sustained atrial tachyarrhythmia was defined as atrial flutter, atrial tachycardia, or AF lasting for more than 30 s in Holter monitoring or more than 10 seconds in 12-lead EKG. LR was defined as sustained atrial tachyarrhythmia within 1 year of RFCA, but early recurrence during the blanking period of 3 months after ablation was not regarded as late recurrence. The AF duration was defined as the difference between the date of the first AF documented on EKG and the date of index RFCA. The eGFR was calculated using the CKD-EPI (chronic kidney disease–epidemiology collaboration) equation as follows: eGFR = 141 × min(Scr/*κ*, 1)^*α*^ × max(Scr/*κ*, 1)^−1.209^ × 0.993^Age^ × 1.018 [if woman] × 1.159 [if black], where Scr is the serum creatinine level, *κ* is 0.7 for women and 0.9 for men, *α* is −0.329 for women and −0.411 for men, and min and max denote the minimum and maximum between their arguments, respectively [21].

### 2.6. Dataset Preparation

**Figure 1:**
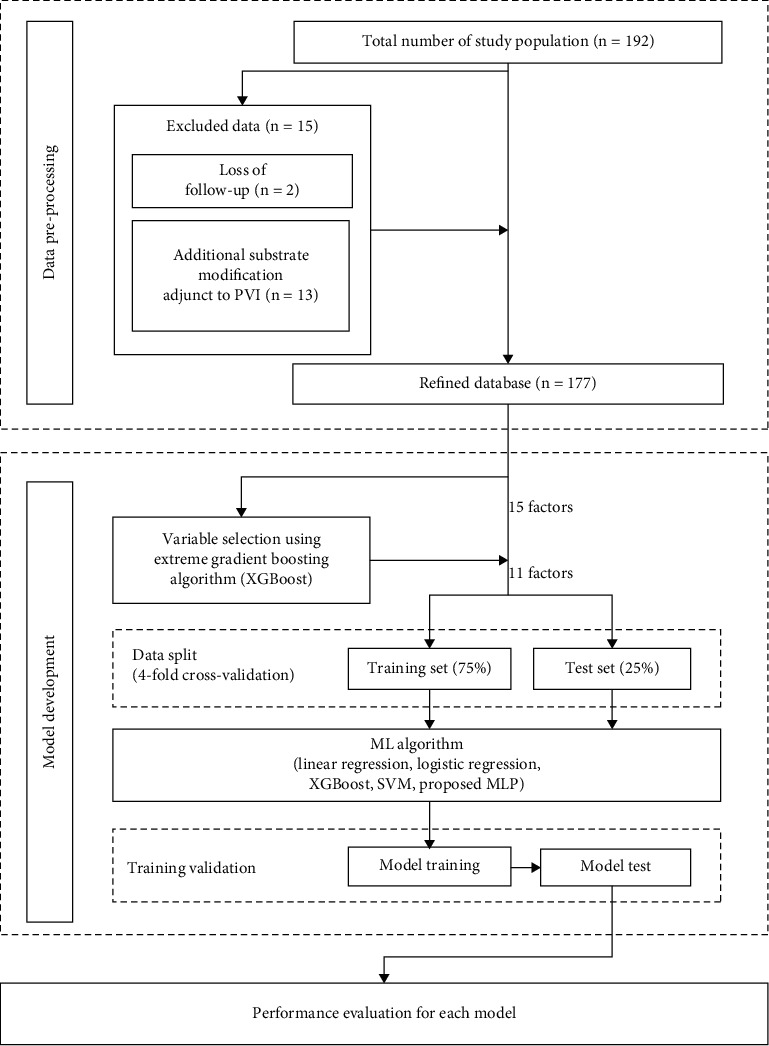
Flowchart of the experimental procedure. Of the 15 factors, 11 were selected using the extreme gradient boosting (XGBoost) algorithm. We used 4-fold cross-validation for model evaluation and applied the synthetic minority oversampling technique to the training set in each fold. ML, machine learning; MLP, multilayer perceptron; PVI, pulmonary vein isolation.

For a small dataset, selecting informative features is essential to ensure training stability and convergence. Thus, extreme gradient boosting (XGBoost) was utilized to select informative features while omitting the irrelevant ones according to the weight of each feature during analysis (Figure 1).

In our dataset, only 47 patients (26.5%) experienced LR. To address the data imbalance that drastically degrades ML performance, we adopted the synthetic minority oversampling technique [22] for data augmentation according to the neighborhood of the minority class. Note that this technique was applied to the training set but not to the test set.

We performed 4-fold cross-validation on our dataset. The dataset was split into 4 equally sized folds. Specifically, in each iteration, one-fold was used for testing (25%) and the others were used for model training (75%).

**Figure 2:**
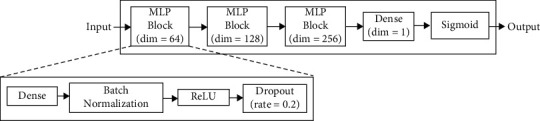
Network architecture based on multilayer perceptron (MLP) to predict the late recurrence probability. Dim, dimension; ReLU, rectified linear unit.

Figure 2 shows the proposed deep learning model based on the MLP architecture. The model consists of three MLP blocks and one output layer. Each MLP block comprises a dense layer, a batch normalization layer, rectified linear unit (ReLU) activation, and a dropout layer with a rate of 0.2 to avoid overfitting. For the output layer, a dense layer followed by sigmoid activation is used to calculate the LR probability. We use weighted binary cross-entropy as the loss function to handle data imbalance. In this study, the model was trained for 1,000 epochs using the Adam optimizer with a learning rate of 10^−4^.

### 2.7. Evaluation of Model Performance

We compared the performance of the proposed model with that of various ML techniques: linear regression, logistic regression, XGBoost algorithm, and SVM. The proposed model and ML techniques were implemented and evaluated using the Keras and TensorFlow 2 platforms in Python 3.8. The proposed model was trained on the CUDA 11.0.3 toolkit using an NVIDIA GeForce RTX 3090 graphics processor.

For the quantitative evaluation, we determined the AUROC, F1 score, sensitivity, and specificity. The receiver operating characteristic (ROC) curve is a statistical performance measure that depicts the true positive rate according to the false positive rate. The AUROC ranges between 0 and 1, with 0.5 indicating random guessing and 1 indicating perfect classification. The F1 score ranges from 0 to 1 and is the harmonic mean of the precision and recall. The accuracy indicates the similarity between measured and actual values, being an intuitive indicator of model performance. The sensitivity is a measure of the true positive rate, and the specificity is a measure of the true negative rate.

### 2.8. Statistical Analysis

Categorical features were compared using Pearson's *χ*^2^ test or Fisher's exact test when the numbers were below five. The normality of continuous features was evaluated using the Shapiro–Wilk test. The difference for continuous features with normal distribution was compared using Student's *t*-test, and distributions with skewed features were compared using the Mann–Whitney test. The ROC curves were plotted with the AUROC to evaluate the diagnostic accuracy of the APPLE and CHA_2_DS_2_-VASc score for LR after the procedure. All the statistical analyzes were performed using SPSS version 28.0 (IBM, Armonk, NY, USA). We compared the performance of the proposed model with that of the conventional APPLE and CHA_2_DS_2_-VASc scores.

## 3. Results

### 3.1. Patient Characteristics

Fifteen of the 192 consecutive patients were excluded because of follow-up loss (*n* = 2) or additional substrate modification adjunct to PVI (*n* = 13). Of the remaining 177 patients, 47 (26.5%) experienced LR within 1 year after RFCA. LR was identified in 17 (19.1%) of 89 patients with paroxysmal AF and 30 (34.1%) of 88 patients with persistent AF. The baseline characteristics of patients with and without AF following RFCA are summarized in Table 1. Patients with LR had higher APPLE scores (1 (0–2) vs. 1 (1–2), *p* = 0.026) and proportion of embolism events (8.5% vs. 19%, *p* = 0.047) than those without LR. A higher proportion of women (12% vs. 23%, *p* = 0.070) and LVEF below 50% (2.3% vs. 8.5%, *p* = 0.082), large LA (41 ± 6 vs. 43 ± 7 mm, *p* = 0.090), and low eGFR (95 ± 21 vs. 88 ± 22, *p* = 0.055) were observed in patients with LR compared with those without LR.

### 3.2. Feature Selection Using Extreme Gradient Boosting

**Figure 3:**
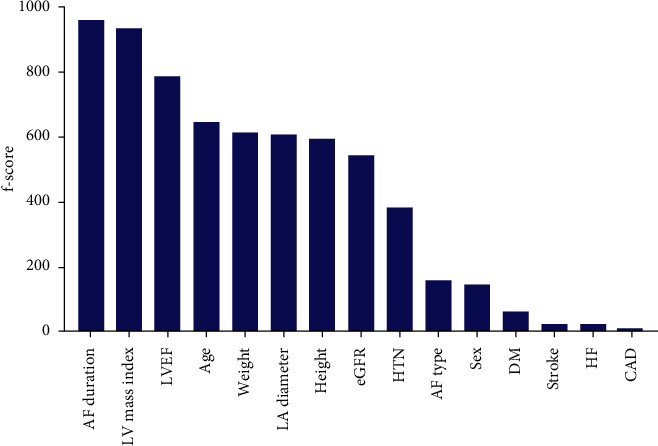
Importance of features obtained from extreme gradient boosting algorithm.AF, atrial fibrillation; LV, left ventricular; LVEF, left ventricular ejection fraction; LA, left atrium; eGFR, estimated glomerular filtration rate; HTN, hypertension; AF type, paroxysmal AF vs. persistent AF; DM, diabetes mellitus; HF, heart failure; CAD, coronary artery disease.

Figure 3 shows the relative importance of risk factors for LR following RFCA. We selected representative features with an f-score higher than 100 as input of the proposed model: age, sex, height, weight, hypertension, AF type, AF duration, LA diameter, left ventricular mass index, LVEF, and eGFR.

### 3.3. Performance Evaluation

**Figure 4:**
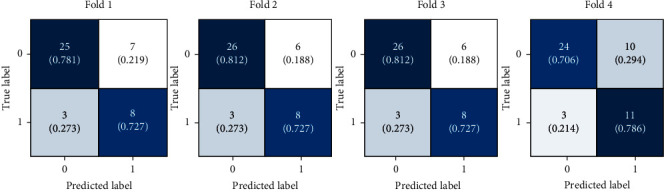
Confusion matrices of the proposed model for 4-fold cross-validation.

**Figure 5:**
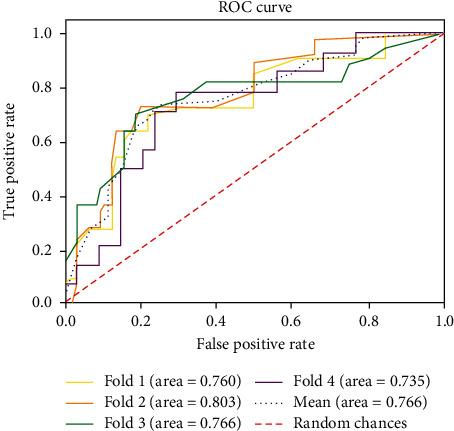
Receiver operating characteristic (ROC) curves for late recurrence of atrial fibrillation after radiofrequency catheter ablation obtained from the proposed model stratified over 4-fold cross-validation.

To evaluate the model performance, we performed 4-fold cross-validation on our dataset. The resulting AUROC, F1 score, sensitivity, specificity, and accuracy per iteration of the proposed model are listed in Table 2. Each fold showed AUROC ≥ 0.73.  Figure 4 shows the confusion matrix for every fold, and Figure 5 shows the ROC curves for the 4-fold average and all folds.

**Figure 6:**
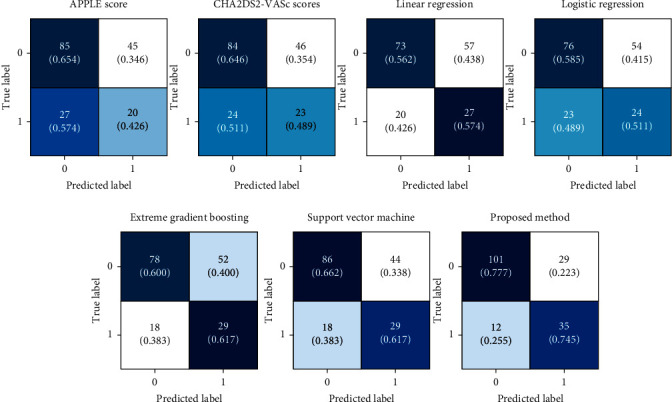
Confusion matrices of conventional prediction models and machine learning approaches. APPLE, acute patient physiologic and laboratory evaluation.

**Figure 7:**
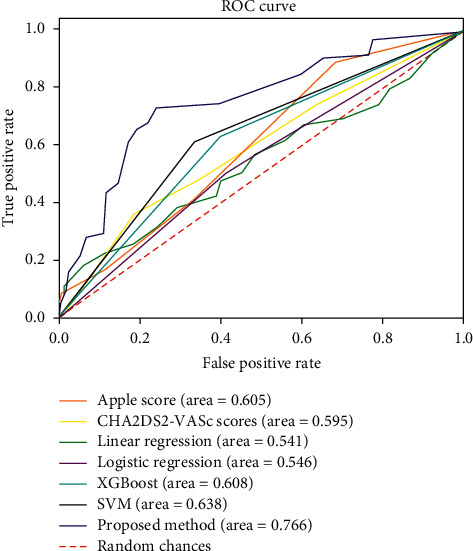
Receiver operating characteristic (ROC) curves for late recurrence of atrial fibrillation after radiofrequency catheter ablation obtained from conventional prediction models and machine learning approaches. APPLE, acute patient physiologic and laboratory evaluation; XGBoost, extreme gradient boosting; SVM, support vector machine.

Table 3 shows the quantitative evaluation results for the evaluated models. The APPLE and CHA_2_DS_2_-VASc score underperformed the ML techniques (i.e., linear regression, logistic regression, XGBoost algorithm, and SVM).  Figure 6 shows the confusion matrices for all the evaluated models, and Figure 7 shows the ROC curves. Our model achieves an average AUROC of 0.766, F1 score of 0.632, sensitivity of 0.745, and specificity of 0.777. The model showed the highest performance compared to those of the conventional prediction models and ML approaches.

## 4. Discussion

Our results demonstrate that an MLP-based model using easily accessible clinical and echocardiographic features obtained during the preprocedural stage can suitably predict LR after RFCA. Our model outperformed conventional prediction models and ML approaches. This approach may support decision-making for selecting patients with AF considering the LR probability after RFCA.

### 4.1. Interpretation of the Feature Importance

To observe the impact of each feature on the MLP model training, we utilized Shapley additive planation (SHAP) [23], which is one of the widespread methods to explain model predictions and provide visualization charts. More specifically, the SHAP algorithm calculates the relative importance of the features on the prediction. The magnitude of the SHAP value means the degree of influence on the prediction. The positive SHAP value indicates that the feature contribution to the probability of AF recurrence is higher, and a negative SHAP value indicates that the feature contribution to the probability of AF recurrence is lower.

**Figure 8:**
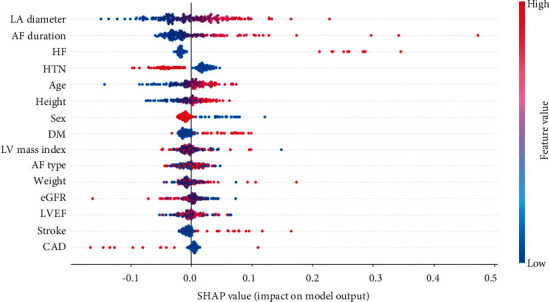
The Shapley additive explanation (SHAP) summary plot of the proposed model. It represents the feature importance of the model output. The color of the dots indicates the attribution value of the feature. For categorical features (CAD {0, 1}; AF type {0: paroxysmal, 1: persistent}; DM {0, 1}; HTN {0, 1}; sex {0: female, 1: male}; stroke {0, 1}; HF {0, 1}), the red and blue dots represent 1 and 0, respectively. AF, atrial fibrillation; LV, left ventricular; LVEF, left ventricular ejection fraction; LA, left atrium; eGFR, estimated glomerular filtration rate; HTN, hypertension; AF type, paroxysmal AF vs persistent AF; DM, diabetes mellitus; HF, heart failure; CAD, coronary artery disease.

As illustrated in Figure 8, the LA diameter was the most powerful feature followed by AF duration, weight, eGFR, and LV mass index. In addition, the SHAP results demonstrated that the higher value of LA diameter, the higher value of AF duration, patients with heart failure, patients with hypertension, the higher value of age, the higher value of height, the lower value of sex (0: female, 1: male), patients with diabetes mellitus, patients with stroke were associated with an increased risk of AF recurrence. Interestingly, some features showed unexpected contributions to the AF recurrence in the SHAP results. The AF type gave a lower influence than AF duration, LV mass index, and LA diameter, while the AF type is widely accepted as the primary risk factor for LR after RFCA [24]. This finding can be explained by the fact that the rhythm outcome after RFCA may differ according to the different burdens of AF, especially paroxysmal AF [38].

### 4.2. Conventional Prediction Models for Radiofrequency Catheter Ablation Prognosis in Patients with Atrial Fibrillation

Clinical risk factors including the AF type and duration [16, 17, 25], obesity [26], sleep apnea [27], and hypertension [28] are associated with the development of abnormal atrial substrate that leads to AF recurrence after RFCA. Moreover, the LA diameter and volume [29], the volume of epicardial fat [30], and the severity of atrial tissue fibrosis [31] are structural predictors of the RFCA outcome in patients with AF. However, no single factor has shown superiority over others in predicting the outcome.

To overcome this limitation, various prediction models combining well-known risk factors have been developed, with models including ALARMEc, HATCH, CHA_2_DS_2_-VASc, and APPLE scores showing a moderate performance with AUROC ranging from 0.44 to 0.74 [32]. However, these scoring models may not reflect high-order interactions between various features because they consider simple linear equations, in which one or two points are arbitrarily assigned to the corresponding risk factors. Although the concordance statistics of the BASE-AF2 score have shown good to excellent discrimination ability of 0.61–0.94 [33], postprocedural features such as early recurrence after RFCA should be included to show such performance. Similarly, the MB-LATER score with AUROC ranging from 0.57 to 0.83 should include early recurrence after RFCA to calculate the scoring system to provide an excellent performance [34].

### 4.3. Machine Learning Models for Radiofrequency Catheter Ablation Prognosis in Patients with Atrial Fibrillation

A deep learning model has shown a good prediction performance (C-index of 0.76) by simply using four features: N-terminal pro brain natriuretic peptide, AF type, LA appendage volume, and LA volume [35]. The easily obtainable input data used in this model may enable practical application. However, this model excludes accepted clinical features including AF duration and comorbidities related to LR after RFCA. More recently, Baalman et al. [36] proposed an ML prediction model using input data selected from 166 clinical features. Although the model suitably predicts LR (AUROC of 0.73, 95% confidence interval of 0.68–0.77), the continuous input features, such as age, LA volume index, and CHA_2_DS_2_-VASc score, are expressed as discrete values, leading to information loss.

Unlike existing ML models, the proposed MLP-based deep learning prediction model uses continuous and multiple features without information loss. Moreover, it can achieve a superior discriminative ability compared with established prediction models such as the APPLE and CHA_2_DS_2_-VASc score. The promising performance of our model may be attributed to the ability of MLP to learn high-order interactions between accepted risk factors and the rhythm outcomes after RFCA.

### 4.4. Limitations of the Study

Various limitations of this study should be noted. First, LR may have been underestimated because the rhythm outcome after RFCA was evaluated by intermittent EKG and Holter monitoring. Second, the study population included in this study was limited. Nevertheless, to prevent overfitting due to the small sample size and evaluate the test model robustness, we employed 4-fold cross-validation, consistently achieving an AUROC above 0.73 in each fold. Third, deviations of the patient's characteristics may have occurred owing to relatively short AF duration, a smaller proportion of heart failure with reduced ejection fraction, and pulmonary vein isolation lesion set at index procedure. Therefore, the model performance should be confirmed by considering an external validation cohort in future work. Finally, we did not consider a recent deep learning model that uses LA fibrosis findings in magnetic resonance images as an input feature [37].

## 5. Conclusion

We proposed an MLP-based model that outperforms conventional prediction models and state-of-the-art ML methods in predicting rhythm outcomes after RFCA in patients with AF. The model may support clinical decision-making for selecting good responders to the RFCA intervention. In future work, we will further to improve the proposed model by considering imaging data related to the atrial substrate.

## Figures and Tables

**Table 1:** Baseline characteristics of patients with and without late recurrence following catheter ablation.

	Without late recurrence (*n* = 130)	With late recurrence (*n* = 47)	*P* value
Age (years)	59 ± 10	60 ± 10	0.626
Age > 65 years	35 (27)	16 (34)	0.356
Female sex	16 (12)	11 (23)	0.070
Height (cm)	167 ± 7	166 ± 8	0.604
Body weight (kg)	72 ± 11	71 ± 12	0.552
BMI (kg/m^2^)	26 ± 3	26 ± 4	0.686
Persistent atrial fibrillation	58 (45)	30 (64)	0.024
AF duration (month)	23 ± 25	36 ± 41	0.054
Heart failure	4 (3)	7 (15)	0.009
Hypertension	49 (38)	13 (28)	0.217
Diabetes mellitus	21 (16)	9 (19)	0.639
Prior stroke or TIA or SE	11 (8.5)	9 (19)	0.047
Vascular disease	8 (6)	4 (9)	0.582
TTE findings			
LA diameter (mm)	41 ± 6	43 ± 7	0.090
LA diameter ≥ 43 mm	49 (38)	23 (49)	0.179
LVEF (%)	69 ± 9	68 ± 13	0.515
LVEF < 50%	3 (2.3)	4 (8.5)	0.082
LV mass index (g/m^2^)	87 ± 19	90 ± 28	0.452
Laboratory findings			
eGFR (ml/min/1.73 m^2^)	95 ± 21	88 ± 22	0.055
eGFR < 60 ml/min/1.73 m^2^	5 (4)	3 (6)	0.359
CHA_2_DS_2_-VASc score, median (IQR)	1 (0–2)	1 (0–2)	0.299
APPLE score, median (IQR)	1 (0–2)	1 (1–2)	0.026

Results are presented as *n* (%) or means with standard deviation. AF, atrial fibrillation; BMI, body mass index; eGFR, estimated glomerular filtration rate; LA, left atrium; LV, left ventricle, LVEF, left ventricular ejection fraction; SE, systemic embolism; TIA, transient ischemic attack; TTE, transthoracic echocardiography.

**Table 2:** Quantitative evaluation results of the proposed model for 4-fold cross-validation.

	AUROC	F1 score	Sensitivity	Specificity	Accuracy
Fold 1	0.760	0.615	0.727	0.781	0.767
Fold 2	0.803	0.640	0.727	0.812	0.791
Fold 3	0.766	0.640	0.727	0.812	0.791
Fold 4	0.735	0.629	0.786	0.706	0.729
Mean	**0.766**	**0.633**	**0.745**	**0.777**	**0.768**

The mean values are indicated in bold.

**Table 3:** Quantitative evaluation results of evaluated models.

	AUROC	F1 score	Sensitivity	Specificity	Accuracy
APPLE score	0.605	0.357	0.426	0.654	0.593
CHA_2_DS_2_-VASc score	0.595	0.397	0.489	0.646	0.605
Linear regression	0.541	0.412	0.574	0.562	0.565
Logistic regression	0.546	0.381	0.511	0.585	0.565
XGBoost	0.608	0.452	*0.617*	0.600	0.605
SVM	*0.638*	*0.482*	*0.617*	*0.662*	*0.650*
Proposed model	**0.766**	**0.632**	**0.745**	**0.777**	**0.768**

The best and second-best results are shown in boldface and italics, respectively.

## Data Availability

The data underlying this article cannot be shared publicly due to ethical issues.
